# Genome-wide association study for flowering time, maturity dates and plant height in early maturing soybean (*Glycine max*) germplasm

**DOI:** 10.1186/s12864-015-1441-4

**Published:** 2015-03-20

**Authors:** Jiaoping Zhang, Qijian Song, Perry B Cregan, Randall L Nelson, Xianzhi Wang, Jixiang Wu, Guo-Liang Jiang

**Affiliations:** Plant Science Department, South Dakota State University, Brookings, SD 57006 USA; Soybean Genomics and Improvement Laboratory, US Department of Agriculture, Agricultural Research Service (USDA-ARS), 10300 Baltimore Ave, Beltsville, MD 20705 USA; USDA-ARS, Soybean/Maize Germplasm, Pathology, and Genetics Research Unit and Department of Crop Sciences, University of Illinois, Urbana-Champaign, 1101 West Peabody Drive, Urbana, IL 61801 USA; Agricultural Research Station, Virginia State University, P.O. Box 9061, Petersburg, VA 23806 USA

**Keywords:** Genetic architecture, Genetic improvement, GWAS, Quantitative trait locus, Single nucleotide polymorphism, Soybean (*Glycine max*)

## Abstract

**Background:**

Soybean (*Glycine max*) is a photoperiod-sensitive and self-pollinated species. Days to flowering (DTF) and maturity (DTM), duration of flowering-to-maturity (DFTM) and plant height (PH) are crucial for soybean adaptability and yield. To dissect the genetic architecture of these agronomically important traits, a population consisting of 309 early maturity soybean germplasm accessions was genotyped with the Illumina Infinium SoySNP50K BeadChip and phenotyped in multiple environments. A genome-wide association study (GWAS) was conducted using a mixed linear model that involves both relative kinship and population structure.

**Results:**

The linkage disequilibrium (LD) decayed slowly in soybean, and a substantial difference in LD pattern was observed between euchromatic and heterochromatic regions. A total of 27, 6, 18 and 27 loci for DTF, DTM, DFTM and PH were detected via GWAS, respectively. The *Dt1* gene was identified in the locus strongly associated with both DTM and PH. Ten candidate genes homologous to *Arabidopsis* flowering genes were identified near the peak single nucleotide polymorphisms (SNPs) associated with DTF. Four of them encode MADS-domain containing proteins. Additionally, a pectin lyase-like gene was also identified in a major-effect locus for PH where LD decayed rapidly.

**Conclusions:**

This study identified multiple new loci and refined chromosomal regions of known loci associated with DTF, DTM, DFTM and/or PH in soybean. It demonstrates that GWAS is powerful in dissecting complex traits and identifying candidate genes although LD decayed slowly in soybean. The loci and trait-associated SNPs identified in this study can be used for soybean genetic improvement, especially the major-effect loci associated with PH could be used to improve soybean yield potential. The candidate genes may serve as promising targets for studies of molecular mechanisms underlying the related traits in soybean.

**Electronic supplementary material:**

The online version of this article (doi:10.1186/s12864-015-1441-4) contains supplementary material, which is available to authorized users.

## Background

Flowering, maturity and plant height in plants are complex traits controlled by internal and external factors. They considerably impact the adaptability, biomass and economic yield in agricultural crops. The genetic signaling pathways of flowering have been well characterized in *Arabidopsis*, a model organism in plant biology. The floral integrator genes *FLOWERING LOCUS T* (*FT*) and *SUPPRESSOR OF OVEREXPRESSION OF CONSTANS 1* (*SOC1*) play a central role in flowering regulation. They promote the expression of a group of floral meristem identity genes such as *APETALA 1* (*AP1*), *LEAFY* (*LFY*) and *CAULIFLOWER* (*CAL*) to initiate the floral transition of the plant [1]. In the floral pathway, upstream of *FT* and *SOC1* are *FLOWERING LOCUS C* (*FLC*) and *CONSTANS* (*CO*), which are two key regulators of floral integrators but in different manners. In *Arabidopsis*, a high level of FLC represses expression of both *FT* and *SOC1*, and it can be released by vernalization treatment or autonomous development process [1]. *CO* is circadian-regulated and involved in the photoperiod pathway through promoting expression of *FT* and *SOC1* [2,3]. Additionally, more genes involved in the inductive photoperiod pathway, the vernalization pathway, the autonomous pathway and the gibberellins pathway have been characterized in *Arabidopsis* [1,4,5].

Soybean (*Glycine max*) is a major crop of agronomic importance grown across a wide range of latitudes from 50°N to 35°S [6]. However, every soybean cultivar adapts to a limited latitudinal region because of photoperiod sensitivity. Days to flowering (DTF) and maturity (DTM) and plant height (PH) are important traits related to soybean adaptability and productivity [7]. DTM represents the entire duration of growth and development, consisting of two periods: DTF and duration of flowering-to-maturity (DFTM). All the four traits are quantitatively inherited in soybean. Previous studies identified nine major-effect loci affecting flowering and maturity in soybean, which have been designated as *E1* to *E8*, and the *J* locus for “long juvenile period” [6]. Of these genes, *E1*, *E2*, *E3* and *E4* have been map-based cloned and functionally characterized. *E1* encodes a nuclear-localized B3 domain-containing protein, which is induced by long day conditions and is inversely related to both *GmFT2a* and *GmFT5a* expression [8], two *FT* orthologs promoting early flowering in soybean [9]. *E2* encodes a homolog of GIGANTEA, which regulates expression of *CO* and *FT* in *Arabidopsis*, and controls soybean flowering through regulating *GmFT2a* but not *GmFT5a* [10]. *E3* and *E4* encode phytochrome A (PHYA) proteins GmPHYA3 and GmPHYA2, respectively [11,12]. Loss-of-function alleles of *E1*, *E3* or *E4* lead to photoperiod insensitivity and promote early flowering under long day conditions [8,11,12], which are important for soybean plants, a typical short-day crop, to adapt to high-latitude environments. In addition to these major loci, many minor-effect quantitative traits loci (QTLs) related to soybean flowering and maturity have also been identified (SoyBase, www.soybase.org). Recently, comparative genomic analyses revealed that there are a large number of soybean orthologs of *Arabidopsis* flowering genes [6,13], suggesting a complex genetic basis of flowering and maturity in soybean.

Overlaps between PH loci and maturity loci have been observed in soybean [14,15], indicating that PH and maturity might share a similar genetic basis to some extent. Previous research showed that stem termination affected both stem elongation and maturity in soybean, and the gene *determinate stem 1* (*Dt1*) plays a primary role in soybean determination [16]. Determinate soybean plants cease stem elongation through initiating floral transition of the shoot apical meristem (SAM) soon after photoperiod-induced floral transition. In *Arabidopsis,* stem elongation is regulated through cell wall component modification, phytohormone- and light-regulated development [17-19]. To date, at least 180 QTLs associated with PH have been reported across all the 20 chromosomes in soybean (SoyBase, www.soybase.org). However, limited knowledge of genes conditioning soybean stem elongation is available.

Genome-wide association study (GWAS) using high-density markers and a population of non-cross-derived lines provides higher mapping resolution than conventional QTL mapping based on cross-derived segregating populations, and enables one to predict or identify causal genes. GWAS has been widely used to dissect complex traits in some major crops, e.g., maize and rice [20-23]. However, there are very few reports of GWAS with high-density single nucleotide polymorphism (SNP) in soybean [24-26]. Thus the application of GWAS in soybean, a highly self-pollinated crop with complex genome structure, remains to be explored, especially for agronomic traits.

To better understand the genetic architecture of DTF, DTM, DFTM and PH in soybean, we conducted a GWAS for these traits in a population consisting of 309 plant introductions (PIs) with 31,045 SNPs. Many new loci and previously reported loci were identified for each trait. Candidate genes with known function or *Arabidopsis* orthologs were also proposed. This study enriches our knowledge of the genetic basis underlying DTF, DTM, DFTM and PH in soybean and provides valuable markers for molecular breeding of soybean.

## Methods

### Plant materials and field trials

Three hundred and nine accessions, obtained from the USDA Soybean Germplasm Collection, were planted in a randomized complete block design with three replications on the Agricultural Research Farms of South Dakota State University at three locations: Aurora (2011AU), Brookings (2012BK) and Watertown (2012WT), SD. According to the Germplasm Resources Information Network (GRIN, http://www.ars-grin.gov/), most of the PIs originate from China, and 90% are maturity group (MG) 0 and the rest are MG 00 (Additional file 1). They mainly adapt to the upper Midwest in the United States and the southern region in Canada.

### Phenotypic evaluation and statistical analysis

DTF and DTM were recorded in the field as the number of days from planting to the date when 50% of the plants in a plot had showed the first flower and when 95% of the pods had ripened as indicated by mature pod color, respectively. DFTM was calculated as the difference between DTM and DTF (or days from flowering to maturity). PH was the average of four measurements per plot, and each measurement was recorded as the length of main stem from the ground to the top extremity of the plant at maturity. The model for the phenotypic trait was *y*_*ijk*_ = *μ* + *g*_*i*_ + *l*_*j*_ + *(gl)*_*ij*_ + *b*_*k(j)*_ + *e*_*ijk*_, where *μ* is the total mean, *g*_*i*_ is the genetic effect of the *i*^*th*^ genotype, *l*_*j*_ is the effect of the *j*^*th*^ environment, *(gl)*_*ij*_ is the interaction effect between the *i*^*th*^ genotype and the *j*^*th*^ environment, *b*_*k(j)*_ is the block effect within the *j*^*th*^ environment, and *e*_*ijk*_ is a random error following N(0, *σ*^*2*^_*e*_). Broad heritability on an entry-mean basis was calculated as *H*^2^ = *σ*^*2*^_*g*_/[*σ*^*2*^_*g*_ + σ^2^_*gl*_/*k* + σ^2^_*e*_/(*rk*)], where *σ*^*2*^_*g*_ is the genotypic variance, *σ*^*2*^_*gl*_ is the genotype by environment interaction variance, *k* is the number of environments, *r* is the number of replications. Estimation of variance components was performed by the varcomp procedure in SAS version 9.3 (SAS Institute, Inc., Cary, NC) with all effects considered to be random. To estimate the proportion of phenotypic variation explained by the mixed linear model (MLM) containing all identified loci, the likelihood-ratio-based R^2^ was calculated for each trait [27].

### Genotyping and quality control

The Illumina Infinium SoySNP50K BeadChip was used to genotype the population as described in a previous study [28], and 42,509 SNPs were identified with a call success rate of 85% or greater. Of them, 61 SNPs that were presented in unanchored sequence scaffolds were excluded from further analyses. The dataset had a missing rate of 0.6%. Markers with missing rate larger than 10% were ruled out and the remaining missing data were imputed using BEAGLE version 3.3.1 with default parameter settings [29,30]. SNPs with a minor allele frequency (MAF) < 5% after imputation were excluded from further analyses as well. Finally, a total of 31,045 SNPs were used for GWAS.

### Linkage disequilibrium estimation

Pairwise LD between markers was calculated as squared correlation coefficient (*r*^*2*^) of alleles using R package synbreed [31]. In light of substantial difference in recombination rate between euchromatic and heterochromatic regions, *r*^*2*^ was calculated separately for the two chromosomal regions. The physical length of euchromatin and heterochromatin on each chromosome were defined as in SoyBase (www.soybase.org). Only *r*^*2*^ for SNPs with pairwise distance less than 10 Mb in either euchromatic or heterochromatic region of each chromosome were used to draw the average LD decay figure by R script using the equation described in a previous study [32]. The LD decay rate of the population was measured as the chromosomal distance where the average *r*^*2*^ dropped to half its maximum value [23].

### Genome-wide association analysis

To minimize the effects of environmental variation, best linear unbiased predictors (BLUPs) of individual lines were calculated for each trait using the R package lme4 [33], and were then used to fit the one-way ANOVA model for naive test (without correction of population structure and familial relatedness) implemented in R 2.15.3 (www.R-project.org) and MLM implemented in the GAPIT R package [34,35]. The latter takes both familial relatedness and population structure into account.

For the naive test, the equation was

$$ y=\mu +X\alpha +e. $$

For the MLM analysis, the equation was

$$ y=\mu +X\alpha +P\beta +Zu+e, $$

where y is the phenotype BLUPs of each line, *μ* is the total mean, *X* is the incidence matrix relating the individuals to the fixed marker effects *α*, *P* is the incidence matrix relating the individuals to the fixed principal component (PC) effects *β*, and *Z* is the incidence matrix relating the individuals to the random group effects *u* obtained from the compression algorithm. The random group effects *u* follows a multivariate normal distribution with mean 0 and variance-covariance matrix *2KV*_*g*_, where *K* is the kinship matrix, and *V*_g_ is the polygenic variance. The random error term *e* follows a multivariate normal distribution with mean 0 and variance-covariance matrix *IV*_e_, where *I* is the identity matrix and *V*_e_ is the error variance component. The optimal number of PCs to be involved in the MLM was determined by Bayesian information criterion of the model (Additional file 2).

For association analysis of DTF and DTM, regular MLM (*K* model) was suggested by GAPIT. For PH, the results between the regular MLM and compressed MLM (cMLM) were very similar, thus a regular MLM (*K* model) was adopted too. While for DFTM, a *K* model was not applicable (Additional file 2) and regular MLM (*K* + *P* model) could not detect significant association, thus cMLM (*K* + *P* model) with a compression level of 1.9 was used as suggested by GAPIT (Additional file 3). The threshold of significance for SNP-trait associations was determined by the false discovery rate (*q*) < 0.05 or the empirical significance level at *P* < 0.001, whichever was more stringent. To assess the empirical significance of SNPs, we performed 1,000 permutations of genome-wide association analyses. Since we could not find appropriate permutation for *K* + *P* model, permutation was conducted for *K* model only, which was used for association analysis of DTF, DTM and PH as described above. For each iteration, the phenotype values and kinship matrix (*K*) in the MLM remained unchanged, while genotypes of each SNP were permuted. Briefly, we shuffled the rows randomly but kept the order of row names unchanged in a genotypic data file where each column represented one SNP and each row represented one germplasm accession. GAPIT was run with the same parameter setting as the original test for each trait. This method preserves the association between the phenotypes and *K* but eliminates the association between the SNP and *P* (Peter J. Bradbury, personal communication). As a result, we applied it to the *K* model only but not to the *K* + *P* model.

### Prediction of candidate genes

Genes annotated in Glyma1.1, Glyma1.0 and NCBI RefSeq gene models in SoyBase (www.soybase.org) were used as the source of candidate genes. The prediction of candidate genes was referred to the following preferences: i) genes of known function in soybean related to the trait under study, ii) genes with function-known orthologs in *Arabidopsis* related to the trait under study, and iii) genes pinpointed by the peak SNPs.

## Results

### Statistics of phenotypes

Analysis of variance indicated that the effects of genotypes, environments, and their interactions were significant for all the four traits except the *G* × *E* interaction for PH (Table 1). Averaged over three environments, all traits showed a large variation, especially for PH where a four-fold difference was observed. The frequency distribution skewed towards early flowering for DTF but approximated a normal distribution for the other three traits (Additional file 4). There were high correlations between different environments, ranging from 0.77 (*P* < 10^−4^) for DFTM between 2011 AU and 2012WT to 0.91 (*P* < 10^−4^) for DTF between 2011 AU and 2012BK. Accordingly, a high heritability (>80%) was estimated for each trait, indicating that genetic effects play a predominant role in the performance of the traits (Table 1). However, the correlations between traits were low to moderate (Additional file 5). Interestingly, DTF was positively correlated to DTM (*r* = 0.34, *P* < 10^−4^) while negatively correlated to DFTM (*r* = −0.47, *P* < 10^−4^).

**Table 1:** **Statistics of days to flowering (DTF), days to maturity (DTM), duration of flowering-to-maturity (DFTM) and plant height (PH) for the germplasm accessions**

**Trait**	**Mean ± SD**	**Range**	***F***_***G***_^**a**^	***F***_***E***_^**a**^	***F***_***GxE***_^**a**^	**Heritability** ^***b***^ **(%)**
DTF (day)	44.2 ± 4.1	38.4 - 57.0	22.7***	94.7***	2.1***	95.6
DTM (day)	102.3 ± 6.4	90.5 - 111.8	16.4***	833.1***	2.2***	94.2
DFTM (day)	58.2 ± 3.6	46.6 - 68.0	12.7***	211.5***	2.4***	92.2
PH (cm)	74.6 ± 12.3	29.4 - 117.7	17.0***	18.8**	0.5	82.8

^a^ *F*_*G*_, *F*_*E*_, and *F*_*GxE*_ represent the *F* value for genotypic, environmental effects and genotype × environment interaction, respectively.

^b^Entry mean-based heritability: *H* ^2^ = *σ* ^*2*^_*g*_/[*σ* ^*2*^_*g*_ + σ^2^_gl_/*k* + σ^2^_e_/(*rk*)], where *σ* ^*2*^_*g*_ is the genotypic variance, *σ* ^*2*^_*gl*_ is the genotype by environment interaction variance, *k* is the number of environments, *r* is the number of replications.

**P < 0.001; ***P < 0.0001.

### Distribution of markers and linkage disequilibrium

**Figure 1:**
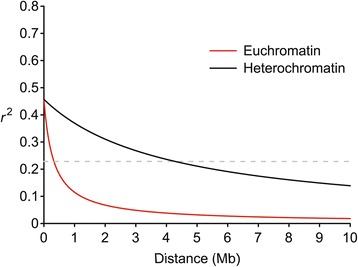
**Average linkage disequilibrium (LD) decay rate in euchromatic and heterochromatic regions of the soybean genome.** The mean LD decay rate was estimated as squared correlation coefficient (*r* ^2^) using all pairs of SNPs located within 10 Mb of physical distance in euchromatic (red) and heterochromatic (black) regions in a population of 309 soybean germplasm accessions. The dashed line in grey indicates the position where *r* ^2^ dropped to half of its maximum value.

A total of 31,045 SNPs with MAF ≥ 0.05 were used for further analyses after quality control, with an average marker density of 1 SNP every 29 kb genome-wide, varying across chromosomes from 44.4 kb/SNP on chromosome 1 (Gm01) to 22.3 kb/SNP on Gm13 (Additional file 6). Most (74.6%) of the SNPs were harbored within euchromatic regions where 78% of putative genes are located [36], resulting in an average marker density of 1 SNP per 20 kb in euchromatin and 1 SNP per 62 kb in heterochromatin. Using the whole set of SNPs, the LD decay rate of the population was estimated at 326 kb in euchromatin, where *r*^*2*^ = 0.23 (half of its maximum value) (Figure 1). In heterochromatin, however, *r*^*2*^ did not drop to half of its maximum value until 4,285 kb.

### GWAS of traits

GWAS was conducted by using the BLUPs of individual performance over three environments in a MLM, which accounts for both population structure and familial relatedness [34,37]. As shown in quantile-quantile plots (Additional file 3), the genomic inflation was considerably controlled in MLMs versus the naive model (i.e., one-way ANOVA model without correcting for relatedness and population structure).

**Figure 2:**
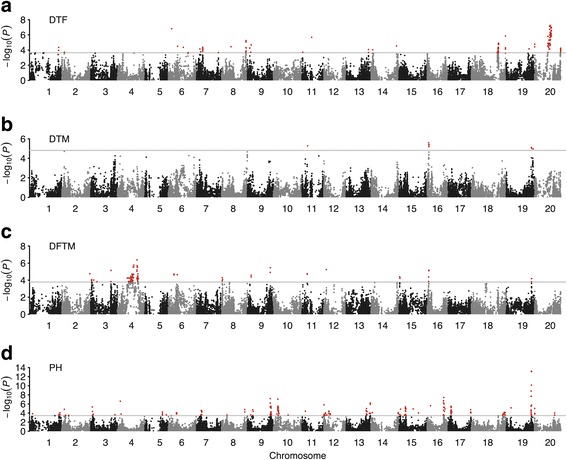
**Manhattan plots of GWAS for each trait in soybean.** Negative log_10_-transformed *P* values from a genome-wide scan by using mixed linear model (MLM) for days to flowering (DTF) **(a)**, maturity (DTM) **(b)** and plant height (PH) **(d)**, and compressed MLM for duration of flowering-to-maturity (DFTM) **(c)** are plotted against positions on each of the 20 chromosomes. The significant trait-associated SNPs (*q* < 0.05) are distinguished by the threshold line and are colored in red.

In total 135, 11, 103 and 115 SNPs significantly associated with DTF, DTM, DFTM and PH were identified, respectively (Figure 2a, b, c and d). Twenty-eight (on Gm18) and 53 (on Gm20) of the 135 SNPs associated with DTF and 66 (on Gm04) of 103 SNPs associated with DFTM were located in the extensive LD blocks in the heterochromatic regions with physical length of 2.9 Mb, 6.3 Mb and 20.0 Mb, respectively (Additional file 7). To determine the trait-associated loci, all significant SNPs located in close physical proximity were clumped at *r*^*2*^ > 0.70 and only the strongest trait-associated SNP (or peak SNP) within each LD block was kept, except in the case of the extensive LD blocks where multiple peak SNPs were found. Finally, 27, 6, 18 and 27 loci associated with DTF, DTM, DFTM and PH were identified across the 20 chromosomes (Additional file 8). The final model containing these loci explained 77.2%, 52.5%, 69.8% and 76.3% of phenotypic variation for DTF, DTM, DFTM and PH, respectively.

**Figure 3:**
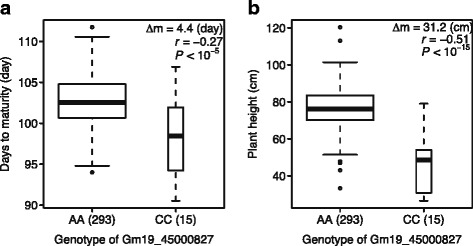
**Phenotypic differences between lines carrying different alleles of the SNP Gm19_45000827 associated with days to maturity (DTM) and plant height (PH).** The boxplot shows the differences of DTM **(a)** and PH **(b)** averaged over three environments between lines with different alleles of the SNP locus. The box shows the first, second (median) and third quartile. The width of the box is proportional to the square root of the number of individuals for each allele. The whiskers extend to the 1.5 times of interquartile or the data extreme whichever is smaller. The number of individual for each allele is given in the parenthesis. The difference of mean (Δm), the Pearson correlation coefficient (*r*) between genotypes and phenotypic values and the *P* value of correlation are also given.

Some loci were found to be associated with multiple traits. The locus at the 45.0 Mb position on Gm19, representing the strongest association for PH, was also associated with DTM. It explained 15% and 4.5% of total phenotypic variation for PH and DTM, respectively. On average, the lines carrying the major frequency allele of the peak SNP (Gm19_45000827, MAF = 0.05) at this locus were 31.2 cm taller and matured 4.4 days later than those with the alternative allele (Figure 3). The locus at the 6.98 Mb position on Gm09 was associated with both DTF and DFTM, but their effects were in opposite directions. Two loci at 2.4 Mb and 3.0 Mb positions on Gm16 were detected for both DTM and DFTM with similar effects. No overlap was found between loci of DTF and DTM.

### Prediction of candidate genes

**Figure 4:**
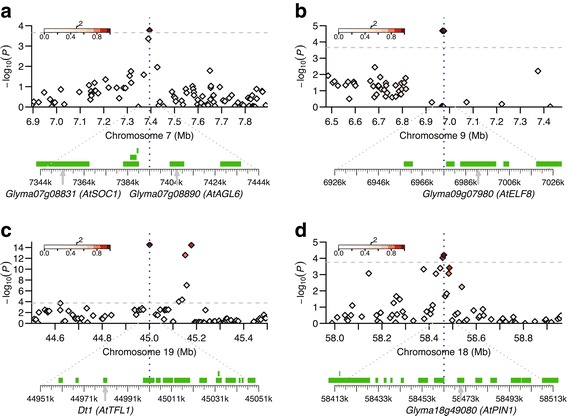
**Candidate genes near the SNP loci associated with days to flowering (DTF) and maturity (DTM) and plant height (PH) in soybean. (a)** and **(b)** Candidate genes for DTF7 and DTF16, respectively; **(c)** Candidate genes for both DTM5 and PH25; and **(d)** Candidate gene for PH21. The top of each panel shows a 0.5-Mb region on each side of the peak SNP, whose position is indicated by a vertical blue dashed line. Negative log10-transformed *P* values from the mixed linear model are plotted on the vertical axis. Significance threshold is indicated as the grey dashed line. The color of each SNP indicates its *r* ^*2*^ value with the peak SNP as shown in the color intensity index on top-left. The bottom of each panel shows all putative genes within the 50 kb adjacent region on each side of the peak SNP as indicated by green boxes. The candidate genes are indicated by arrows and *Arabidopsis* homologs are given in parentheses.

Based on the results of GWAS and genes annotated in SoyBase (www.soybase.org), we further predicted candidate causal genes for loci significantly associated with each trait. A total of 18 candidate genes were predicted for 15 of the 27 loci associated with DTF (Additional file 8). Ten of them have orthologs of *Arabidopsis* flowering genes. *Glyma07g08831* and *Glyma07g08890* were located at 27.8 kb upstream and 8.9 kb downstream of the peak SNP of DTF7 locus, respectively (Figure 4a). *Glyma07g08831* is homologous to *AtSOC1*, and encodes a protein sharing 77% amino acid sequence identity with the product of soybean flowering gene *GmSOC1* [38,39]. *Glyma07g08890* and the candidate gene for DTF26 locus *Glyma20g21082* are both homologous to the *Arabidopsis* flowering gene *AGAMOUS*-*LIKE 6* (*AGL6*) [40] (Additional file 8). The locus at about the 7.0 Mb position on Gm09 associated with DTF was targeted by two SNPs in high LD (*r*^2^ = 0.90). *Glyma09g07980*, identified at 7 kb away from the peak SNP of DTF16 (Figure 4b), is homologous to *Arabidopsis EARLY FLOWERING 8* (*ELF8*) [41].

**Figure 5:**
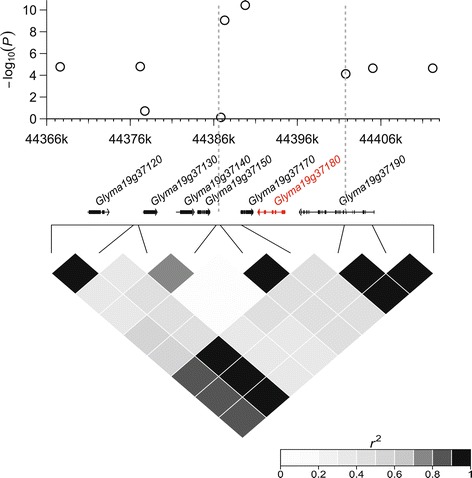
**Candidate range for the major-effect locus PH24 on Gm19 associated with plant height (PH) in soybean.** In the top panel, negative log_10_-transformed *P* values of single nucleotide polymorphisms from a genome-wide association analysis for PH are plotted against physical positions of the given region on Gm19. Bottom panel depicts the extent of linkage disequilibrium in this region based on *r* ^2^. The *r* ^2^ values are indicated using a color intensity index at the right bottom. A region of 15 kb associated with PH24 is indicated by two vertical dashed lines (in grey). Genes within this region are indicated in the middle panel. The proposed candidate gene encoding a putative pectinesterase is highlighted in red.

We identified the *Dt1* gene at 18.6 kb upstream of the peak SNP (Gm19_45000827, MAF = 0.05) on Gm19, which was associated with both DTM and PH (Figure 4c). We also predicted candidate genes for other SNPs associated with PH. A putative gene *Glyma18g49080*, encoding a membrane transport protein, was found at 6.3 kb away from the peak SNP (Gm18_58462762, MAF = 0.22) of locus PH21 on Gm18 (Figure 4d). It is homologous to *Arabidopsis* auxin efflux carrier protein PIN-FORMED 1 (AtPIN1), which is involved in auxin-induced shoot and root development [42]. *Glyma19g37180*, encoding a putative pectinesterase, was identified in a small chromosomal region associated with locus PH24 on Gm19 where LD decayed rapidly (Figure 5). This locus alone could explain 10% of PH phenotypic variation. For DFTM, seven candidate genes were proposed for seven of 18 significantly associated loci. The detailed information of the peak SNPs for all trait-associated loci and candidate genes is presented in the Additional file 8.

## Discussion

Recombination rate is one of the major factors affecting LD extension. In soybean, the recombination rate in euchromatic regions is about five times that in heterochromatic regions [36]. In this study, a large difference in LD decay rate was observed between these two chromosomal regions (326 kb in euchromatin versus 4,285 kb in heterochromatin) (Figure 1). The low LD decay rate in euchromatic regions was also reported in a recent GWAS on soybean [24], in which the population had a higher diversity of origin (China, Korea, Japan) and more maturity groups (II, III and IV) than the population used in this study. It is indicated that the relatively low LD decay rate might be a common phenomenon in Asian soybean landraces, the major source of the USDA Soybean Germplasm Collection. Therefore, LD decay rate is the primary factor limiting the mapping resolution in GWAS for soybean, and a lower density of SNPs should be suitable for GWAS in soybean as compared with other crops like maize and rice.

Kinship and population structure are known as the major confounding factors leading to spurious results in association analysis, and corrected MLM containing both kinship matrix (*K*) and population structure (*P*) is more effective than the MLM containing either *K* or *P* alone [43,44]. In this study, however, no PC was involved in the MLM for the association analysis of DTF, DTM and PH, but two PCs were involved for DFTM (Additional file 2), indicating that the improvement of model fitness by involving *P* in the *K* model could vary with traits. The possible explanations might include: i) the phenotypic variation attributed to population structure varies with traits; and ii) the degree of overlap between *K* and *P* in controlling genetic relationships is different for individual traits [27]. Therefore, the inclusion of population structure in MLM depends on the genetic relationships of the association panel and the divergence of the trait of interest.

Previous reports suggested that DTM and DTF would be highly correlated [45]. However, a low correlation between DTF and DTM (*r* = 0.34, *P* < 10^−4^) was observed in the present study. The relatively higher correlation between DTM and DFTM (*r* = 0.63, *P* < 10^−4^) indicated that DFTM had greater impact on DTM than DTF. In addition, the negative correlation between DTF and DFTM (*r* = −0.47, *P* < 10^−4^) indicated that for a certain early maturity group in soybean, shorter vegetative growth might imply longer reproductive growth to some extent, which may help accumulate more dry matter in seeds. The GWAS results showed that the flowering locus DTF16 was also associated with DFTM but not with DTM, while maturity loci DTM2 and DTM4 were associated with DFTM but not DTF. Therefore, we suppose that some flowering loci may also affect growth stage after flowering as reported in a previous study [46], and some loci condition maturity through affecting reproduction period only. The loci at the 7.0 Mb position on Gm09 for DTF (DFT16) and DFTM (DFTM11) were located within the same region but exhibited opposite effects, which may provide an underlying genetic explanation of the negative correlation between DTF and DFTM.

Previous research showed that major maturity loci (*E1* through *E8*, and *J*) also affected flowering in soybean [6], and *E3* and *E4* affected post-flowering photoperiod responses as well [46]. However, we did not detect these loci for any of the three traits (DTF, DTM and DFTM) in the present study. One possible explanation was lack of functional polymorphism at these loci in the association panel. GWAS using even all SNPs (MAF > 0), including four SNPs in *E2* and one in *E3*, did not detect any of the loci at *q* < 0.05 either. Another explanation was that genetic variants might exist at these major loci, but could not be captured due to the lack of SNP coverage. Notably, some genetic variants were actually undetectable by SNP genotyping. For example, the *e1*-*nl* allele, one of the natural variants of *E1*, was a deletion of the entire gene [8], which is hard to detect through SNP genotyping. The different loci identified between DTF and DTM as well as DFTM suggested that soybean flowering and maturity could be controlled by common major-effect loci, but also modified by numerous trait-specific minor-effect loci.

Natural and artificial selections during domestication can decrease the genetic diversity and increase LD in modern soybean cultivars [47], which difficults prediction of causal genes in soybean through association analysis. In this study, the estimate of genome-wide LD decay rate was much lower than that in rice [23]. However, rapid LD decay was found at some loci, allowing the prediction of candidate genes in these regions (Figures 4 and 5). For DTF, ten of the proposed gene candidates have orthologs of *Arabidopsis* flowering genes, and seven of them were previously identified by comparative genomic analysis [6,13]. Of them, *Glyma07g08831* was located in close proximity to the DTF7 locus. It encodes a protein sharing 77% of amino acid sequence identity with the GmSOC1, which promotes flowering in soybean [38]. The high protein sequence identity between Glyma07g08831 and GmSOC1 might imply their functional abundance in soybean flowering.

The SNP at the 45.0 Mb position on Gm19, a region similar to previously reported QTLs Pod mat 13–6 and Plant height 4–2 and 13–8 (Additional file 8), was strongly associated with both DTM and PH. The *Dt1* gene was found in this region. *Dt1* is homologous to *Arabidopsis terminal flower 1*, and plays a predominant role in determining stem growth habit in soybean [48]. Based upon the stem growth habit, soybean cultivars can be classified into two major categories, determinate and indeterminate. For the determinate soybean cultivars (*dt1/dt1*), SAM switches from vegetative growth to reproductive growth soon after photoperiod-induced floral transition, and stem growth stops [16]. In contrast, the transition of SAM to floral meristem is suppressed in indeterminate cultivars (*Dt1/Dt1*) and vegetative growth of SAM continues until a cessation caused by the demand of developing seeds [49]. Therefore, stem growth habit has broad effects on plant height and maturity of soybean. This is highly consistent with the result of the present study that the locus harboring *Dt1* was strongly associated with both DTM and PH, indicated that *Dt1* is very likely the causal gene for DTM5 and PH25. Because plant height is one of the major factors determining yield potential in soybean, PH25 with large effect on plant height may also affect soybean yield substantially. However, application of the preferred allele of PH25 locus needs to be careful, as it may also affect maturity dates.

The locus PH24, explained 10% of phenotypic variation, was mapped to a small region on Gm19 where LD decayed rapidly. Three QTLs associated with PH were previously reported in the similar region (Additional file 8). The candidate gene *Glyma19g37180* was identified near the peak SNP (Figure 5). It was proposed to encode a pectinesterase (SoyBase, www.soybase.org). Pectin is a structurally complex polysaccharide contained in primary cell walls of plant and has functions in plant growth, morphology and plant defense [50]. Pectinesterase catalyzes the de-esterification of pectin into pectate and methanol, and plays important roles in some physiological processes such as stem elongation that requires rearrangement of cell wall architecture. Transient stem elongation was observed in potato plants overexpressing a *Petunia inflate* pectinesterase [51]. In *Arabidopsis,* a reduction in cross-linking of cell wall pectic polysaccharide resulted in dwarf phenotype [17]. More recent research showed that pectinesterase regulates cell growth and hypocotyl elongation in *Arabidopsis* by affecting the degree of pectin methyl-esterification [52]. Therefore, *Glyma19g37180* encoding a putative pectinesterase was the most likely causal gene for PH24, a major-effect locus associated with plant height in soybean. Notably, unlike PH25, PH24 had no association with maturity, and selection of the desired allele of this locus might improve the yield potential of soybean without affecting the maturity dates.

## Conclusions

In this study, 27, 6, 18 and 27 loci associated with DTF, DTM, DFTM and PH were identified via GWAS, respectively. Thirty-five candidate genes were proposed, including a function-known gene (*Dt1*) and 16 genes orthologous to *Arabidopsis* genes functioning in similar traits. It evidently demonstrates the high efficiency of GWAS in dissecting complex traits in soybean. A medium number of SNPs generated from the SoySNP50K analysis is capable of capturing genome-wide allelic variation, and candidate genes are regionally accessible for crops like soybean with a low LD decay rate. The genetic variants and trait-associated SNPs identified in this study will be useful for soybean cultivar improvement, especially for major-effect loci associated with PH that may have great potential for soybean yield improvement. Additionally, biological validation of the candidate genes will be also of great interest.

## Additional files

Additional file 1:
**List of 309 soybean germplasm accessions analyzed in this study.** Information given in this file for each accession includes accession name, origin and maturity group according to GRIN (http://www.ars-grin.gov/) and the present study. Additional file 2:
**Bayesian Information Criterion (BIC) values of mixed linear model with different numbers of principal components (PCs) used for associate analyses of each trait.**
Additional file 3:
**Quantile-quantile plots of genome-wide association analysis for each trait with different models.** For days to flowering **(a)** and maturity **(b)**, the naive model and regular mixed model (MLM) are presented. For duration of flowering-to-maturity **(c)** and plant height **(d)**, the naive model, MLM and compressed MLM (cMLM) are presented. The expected distribution of negative log_10_-transformed *P* values is indicated in red, and those of the naive model, MLM and/or cMLM are indicated in black, green and/or blue, respectively. Additional file 4:
**Frequency distribution of observations of four agronomic traits in soybean. (a)** Days to flowering (DTF), **(b)** Days to maturity (DTM), **(c)** Duration of flowering-to-maturity (DFTM) and **(d)** Plant height (PH). Shown is the average of each trait in a population of 309 germplasm accessions over 3 environments each with 3 replicates. Additional file 5:
**Correlation analyses of traits.** Information given in this file includes the correlation coefficients of each pair of traits calculated by using the average of each trait over three environments. Additional file 6:
**Distribution and density of single nucleotide polymorphisms (SNPs) across the soybean genome.** Each chromosome is labeled on the horizontal axis and the physical length of each chromosome is labeled on vertical axis. The vertical bar on each chromosome represents the heterochromatic region. The number of SNPs per 100 kb in the consensus data set is shown in a grey scale on right. Additional file 7:
**Extensive linkage disequilibrium (LD) blocks on heterochromatic regions of the soybean genome associated with days to flowering (DTF) and duration of flowering-to-maturity (DFTM). (a)** and **(b)** LD blocks associated with DTF on Gm18 and Gm20, respectively. **(c)** LD block associated with DFTM on Gm04. At the top of each panel, the negative log_10_-transformed *P* values from the regular mixed linear model (MLM) or compressed MLM are plotted against the physical distance on the horizontal axis. The physical length of each region is labeled. In the bottom of each panel, pairwise LD *r* ^*2*^ values are indicated in a low diagonal matrix heat map. The *r* ^*2*^ values are shown using a color intensity index as indicated on right bottom of each panel. Additional file 8:
**List of loci significantly associated with days to flowering (DTF) and maturity (DTM), duration of flowering-to-maturity (DFTM) and plant height (PH) in soybean.** Information given in this file includes the name, alleles, minor allele frequency (MAF), allelic effect and *P* value of genome-wide association analyses of the peak SNP for each locus. The candidate gene(s) and previously reported QTLs at similar chromosome region with some loci identified in this study are also presented.

## Abbreviations

PH: Plant height
BLUP: Best linear unbiased predictor
DFTM: Duration of flowering-to-maturity
DTF: Days to flowering
DTM: Days to maturity
GWAS: Genome-wide association study
MAS: Marker-assisted selection
MG: Maturity group
MAF: Minor allele frequency
MLM: Mixed linear model
PI: Plant introduction
PC: Principal-component
QTL: Quantitative traits locus
SAM: Shoot apical meristem
SNP: Single nucleotide polymorphism
