# Supplementary material for: Genome-wide association study for flowering time, maturity dates and plant height in early maturing soybean (Glycine max) germplasm
Source: BMC Genomics. 2015 Mar 20;16(1):217. doi: 10.1186/s12864-015-1441-4 (PMC4449526; doi:10.1186/s12864-015-1441-4)
Supplement: Additional file 2: — Bayesian Information Criterion (BIC) values of mixed linear model with different numbers of principal components (PCs) used for associate analyses of each trait. [file 12864_2015_1441_MOESM2_ESM.pdf]

Additional file 2: Bayesian Information Criterion (BIC) values of mixed linear model with different numbers of principal components (PCs) used for associate analyses of each trait.

No. of PCs	BIC[a]			
	DTF	DTM	DFTM	PH
0	-671.66	-746.05	-722.88	-1158.49
1	-674.39	-748.81	-725.36	-1161.30
2	-674.58	-750.75	-718.43	-1163.48
3	-676.76	-753.00	-721.27	-1166.30
4	-678.61	-755.87	-722.78	-1162.13
5	-681.00	-756.79	-725.55	-1163.72
6	-683.81	-758.82	-728.40	-1165.60
7	-686.61	-761.45	-730.60	-1168.45
8	-689.36	-763.97	-732.31	-1171.28
9	-689.32	-763.75	-735.05	-1171.49
10	-691.25	-766.58	-733.21	-1174.35

DTF, days to flowering; DTM, days to maturity; DFTM, duration of flowering-to-maturity; PH, plant height.
[a] Larger is better.
