# Supplementary material for: Genome-wide association study for flowering time, maturity dates and plant height in early maturing soybean (Glycine max) germplasm
Source: BMC Genomics. 2015 Mar 20;16(1):217. doi: 10.1186/s12864-015-1441-4 (PMC4449526; doi:10.1186/s12864-015-1441-4)
Supplement: Additional file 3: — Quantile-quantile plots of genome-wide association analysis for each trait with different models. For days to flowering (a) and maturity (b), the naive model and regular mixed model (MLM) are presented. For duration of flowering-to-maturity (c) and plant height (d), the naive model, MLM and compressed MLM (cMLM) are presented. The expected distribution of negative log10-transformed P values is indicated in red, and those of the naive model, MLM and/or cMLM are indicated in black, green and/or blue, respectively. [file 12864_2015_1441_MOESM3_ESM.pdf]

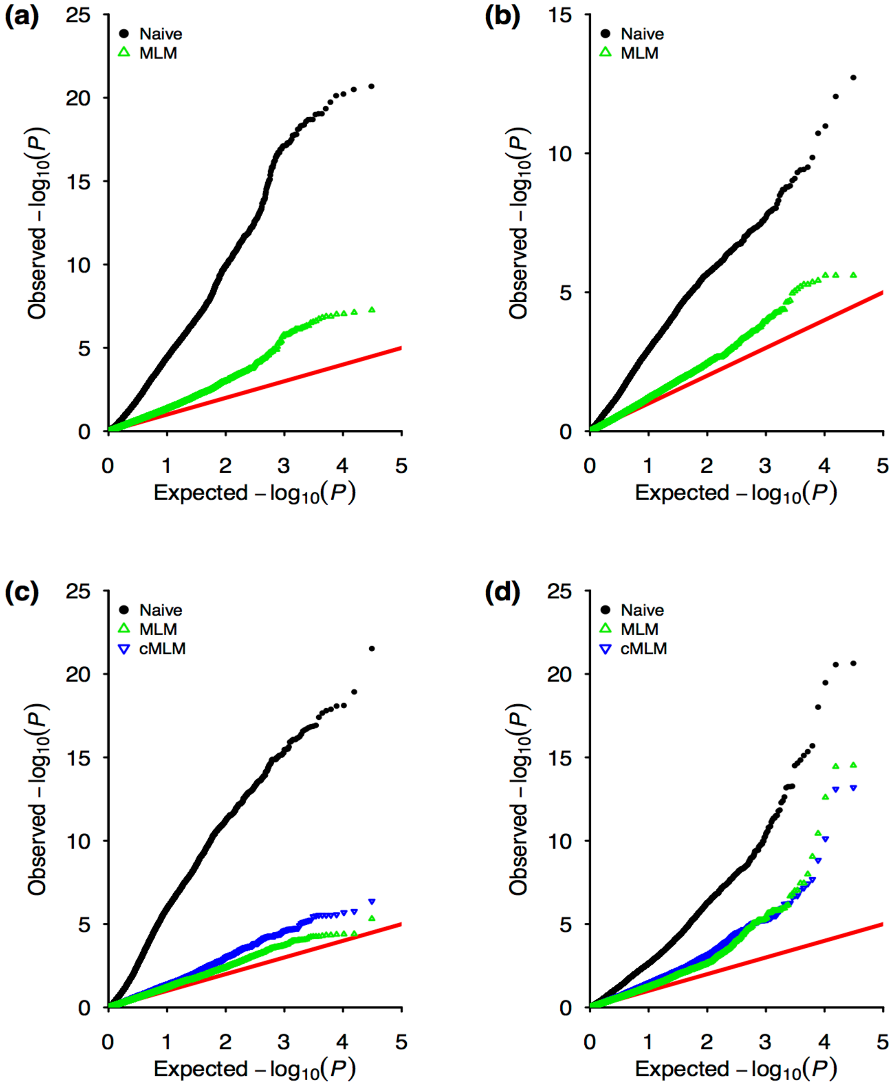

Additional file 3: Quantile-quantile plots of genome-wide association analysis for each trait with different models. For days to flowering (a) and maturity (b), the naive model and regular mixed model (MLM) are presented. For duration of flowering-to-maturity (c) and plant height (d), the naive model, MLM and compressed MLM (cMLM) are presented. The expected distribution of negative $\log_{10}$-transformed P values is indicated in red, and those of the naive model, MLM and/or cMLM are indicated in black, green and/or blue, respectively.
