# Supplementary material for: Genome-wide association study for flowering time, maturity dates and plant height in early maturing soybean (Glycine max) germplasm
Source: BMC Genomics. 2015 Mar 20;16(1):217. doi: 10.1186/s12864-015-1441-4 (PMC4449526; doi:10.1186/s12864-015-1441-4)
Supplement: Additional file 4: — Frequency distribution of observations of four agronomic traits in soybean. (a) Days to flowering (DTF), (b) Days to maturity (DTM), (c) Duration of flowering-to-maturity (DFTM) and (d) Plant height (PH). Shown is the average of each trait in a population of 309 germplasm accessions over 3 environments each with 3 replicates. [file 12864_2015_1441_MOESM4_ESM.pdf]

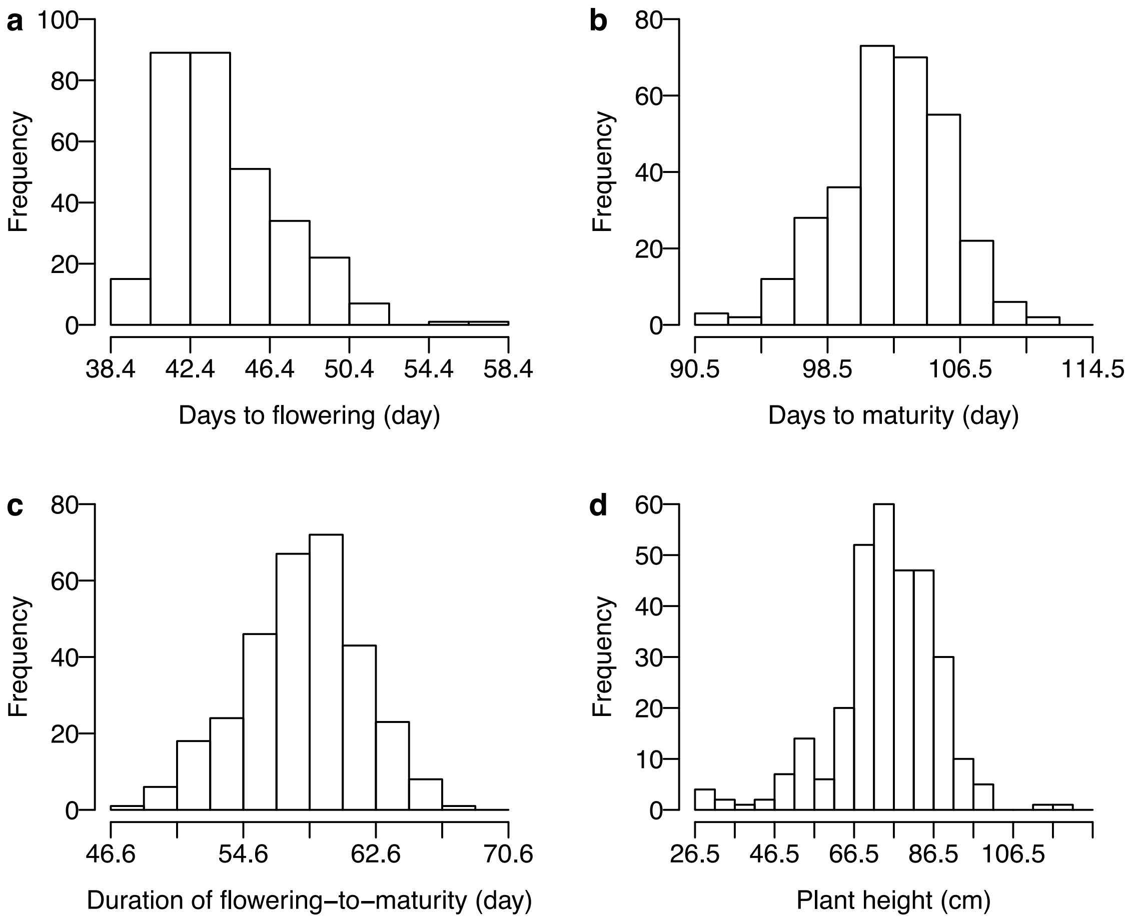

Additional file 4: Frequency distribution of observations of four agronomic traits in soybean. (a) Days to flowering, (b) Days to maturity, (c) Duration of flowering-to-maturity and (d) Plant height. Shown is the average of each trait in a population of 309 germplasm accessions over 3 environments each with 3 replicates.
