# Supplementary material for: Genome-wide association study for flowering time, maturity dates and plant height in early maturing soybean (Glycine max) germplasm
Source: BMC Genomics. 2015 Mar 20;16(1):217. doi: 10.1186/s12864-015-1441-4 (PMC4449526; doi:10.1186/s12864-015-1441-4)
Supplement: Additional file 5: — Correlation analyses of traits. Information given in this file includes the correlation coefficients of each pair of traits calculated by using the average of each trait over three environments. [file 12864_2015_1441_MOESM5_ESM.pdf]

Additional file 5: Correlation coefficients of each pair of traits[a].

	DTF	DTM	DFTM
**DTM**	0.34*		
**DFTM**	-0.47*	0.63*	
**PH**	0.26*	0.54*	0.3*

[a] Data averaged over three environments was used to calculate the correlation coefficients.

* Significant at $P < 10^{-4}$

DTF, days to flowering; DTM, days to maturity; DFTM, duration from flowering to maturity; PH, plant height.
