# Supplementary material for: Genome-wide association study for flowering time, maturity dates and plant height in early maturing soybean (Glycine max) germplasm
Source: BMC Genomics. 2015 Mar 20;16(1):217. doi: 10.1186/s12864-015-1441-4 (PMC4449526; doi:10.1186/s12864-015-1441-4)
Supplement: Additional file 6: — Distribution and density of single nucleotide polymorphisms (SNPs) across the soybean genome. Each chromosome is labeled on the horizontal axis and the physical length of each chromosome is labeled on vertical axis. The vertical bar on each chromosome represents the heterochromatic region. The number of SNPs per 100 kb in the consensus data set is shown in a grey scale on right. [file 12864_2015_1441_MOESM6_ESM.pdf]

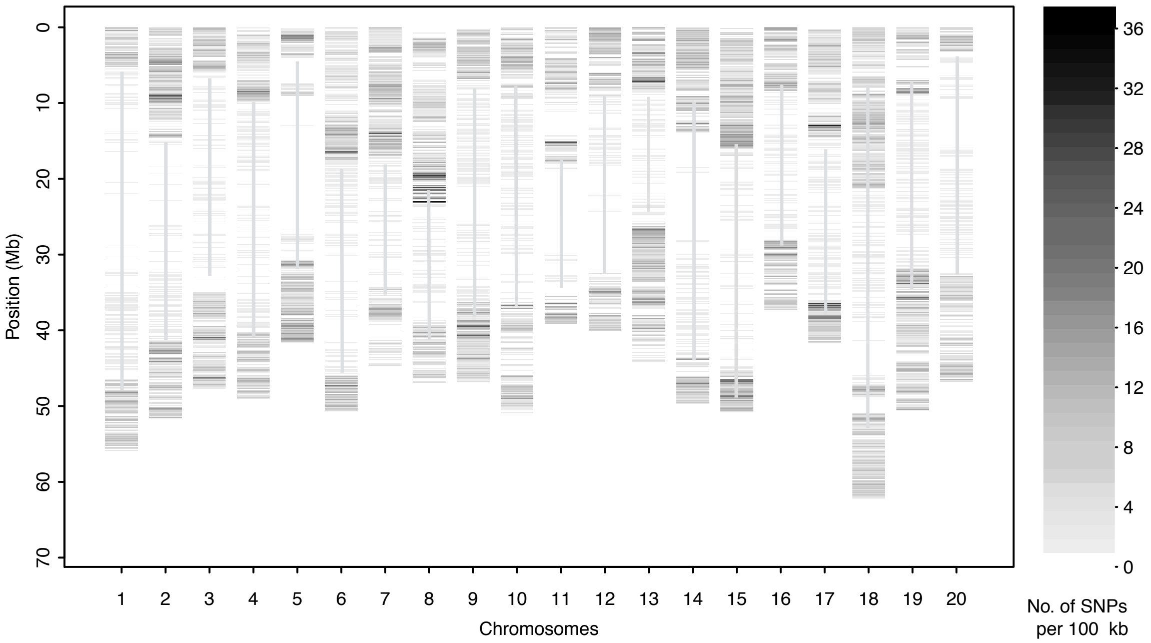

Additional file 6: Distribution and density of single nucleotide polymorphisms (SNPs) across the soybean genome. Each chromosome is labeled on the horizontal axis and the physical length of each chromosome is labeled on vertical axis. The vertical bar on each chromosome represents the heterochromatic region. The number of SNPs per 100 kb in the consensus data set is shown in a grey scale on right.
