# Supplementary material for: Genome-wide association study for flowering time, maturity dates and plant height in early maturing soybean (Glycine max) germplasm
Source: BMC Genomics. 2015 Mar 20;16(1):217. doi: 10.1186/s12864-015-1441-4 (PMC4449526; doi:10.1186/s12864-015-1441-4)
Supplement: Additional file 7: — Extensive linkage disequilibrium (LD) blocks on heterochromatic regions of the soybean genome associated with days to flowering (DTF) and duration of flowering-to-maturity (DFTM). (a) and (b) LD blocks associated with DTF on Gm18 and Gm20, respectively. (c) LD block associated with DFTM on Gm04. At the top of each panel, the negative log10-transformed P values from the regular mixed linear model (MLM) or compressed MLM are plotted against the physical distance on the horizontal axis. The physical length of each region is labeled. In the bottom of each panel, pairwise LD r 2 values are indicated in a low diagonal matrix heat map. The r 2 values are shown using a color intensity index as indicated on right bottom of each panel. [file 12864_2015_1441_MOESM7_ESM.pdf]

(a)

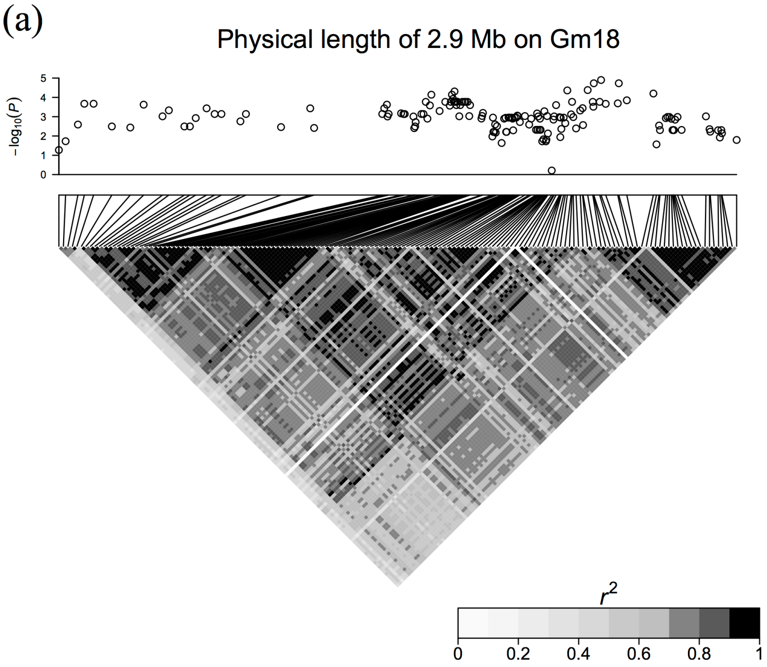
Physical length of 2.9 Mb on Gm18
−log10(P)
r2
0
0.2
0.4
0.6
0.8
1

(b)

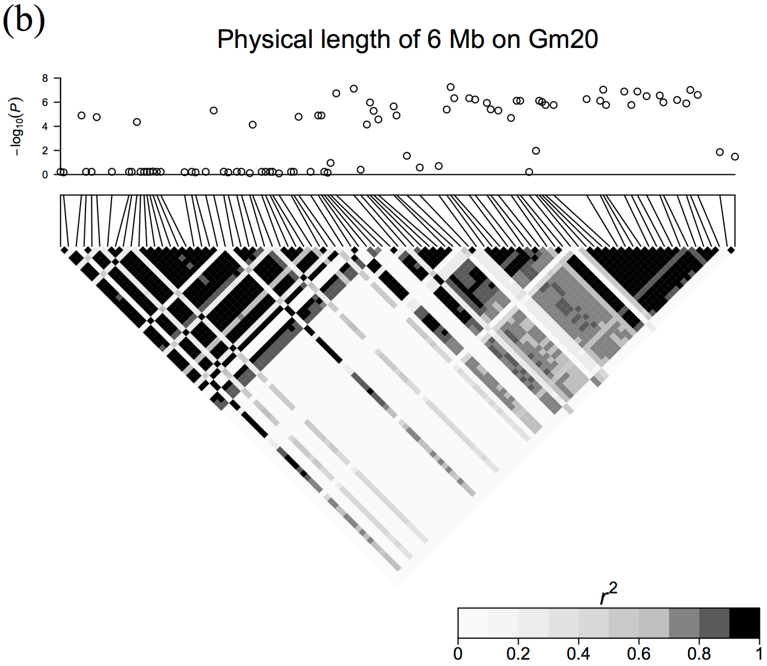
Physical length of 6 Mb on Gm20
−log10(P)
r2
0
0.2
0.4
0.6
0.8
1

(c)

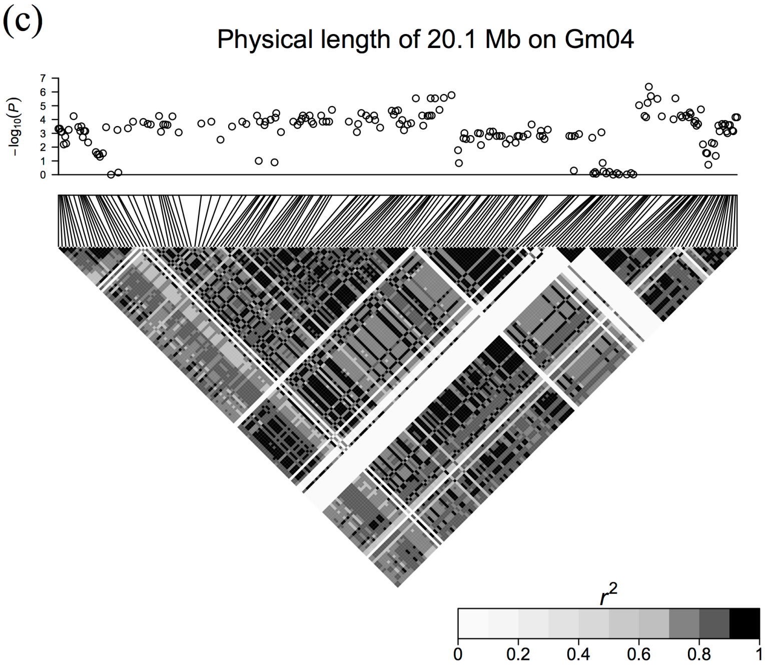

Additional file 7: Extensive linkage disequilibrium (LD) blocks on heterochromatic regions of the soybean genome associated with days to flowering (DTF) and duration of flowering-to-maturity (DFTM). (a) and (b) LD blocks associated with DTF on Gm18 and Gm20, respectively. (c) LD block associated with DFTM on Gm04. At the top of each panel, the negative $\log_{10}$-transformed *P* values from the regular mixed linear model (MLM) or compressed MLM are plotted against the physical distance on the horizontal axis. The physical length of each region is labeled. In the bottom of each panel, pairwise LD r^2 values are indicated in a low diagonal matrix heat map. The r^2 values are shown using a color intensity index as indicated on right bottom of each panel.
