# Supplementary material for: Genome-wide association study for flowering time, maturity dates and plant height in early maturing soybean (Glycine max) germplasm
Source: BMC Genomics. 2015 Mar 20;16(1):217. doi: 10.1186/s12864-015-1441-4 (PMC4449526; doi:10.1186/s12864-015-1441-4)
Supplement: Additional file 8: — List of loci significantly associated with days to flowering (DTF) and maturity (DTM), duration of flowering-to-maturity (DFTM) and plant height (PH) in soybean. Information given in this file includes the name, alleles, minor allele frequency (MAF), allelic effect and P value of genome-wide association analyses of the peak SNP for each locus. The candidate gene(s) and previously reported QTLs at similar chromosome region with some loci identified in this study are also presented. [file 12864_2015_1441_MOESM8_ESM.pdf]

Additional file 8: List of loci significantly associated with days to flowering (DTF) and maturity (DTM), duration of flowering-to-maturity (DFTM) and plant height (PH) in soybean.

Traits	Loci	SNP[a]	Alleles (minor : major)	MAF[b]	Allelic effect[c] (day)	*P*-value	Candidate genes[d]	Annotation	Known-loci[e]
DTF	1	Gm01_50219219	A:C	0.08	1.2	4.5E-05	*Glyma01g37980*	Late embryogenesis abundant protein	
	2	Gm02_4465606	T:C	0.30	0.7	1.9E-04	*Glyma02g05580*	Zinc finger protein	
	3	Gm06_6074331	G:A	0.12	1.4	1.5E-07	*Glyma06g08270*	AIG2-like	
	4	Gm06_16723946	G:A	0.15	1.1	3.2E-05	*Glyma06g20310*	AtBUC2 [1]	Fflr 18-2, Ft 5-1
	5	Gm06_26933523	T:C	0.09	1.2	4.4E-05			Ft 4-2, 5-1, FT 2-1, Fflr 12-2, 4-1, 5-1, 9-1
	6	Gm07_5957056	G:A	0.17	0.9	7.1E-05			Fflr 2-2
	7	Gm07_7394287	T:C	0.17	0.8	1.7E-04	*Glyma07g08890* *Glyma07g08831*	AtAGL6 [2], GmSOC1 [3]	Fflr 2-2
	8	Gm07_11068398	G:A	0.36	-0.8	4.3E-05			Fflr 2-2
	9	Gm07_38991567	G:A	0.31	0.6	2.1E-04			
	10	Gm08_17819435	C:T	0.18	1.0	3.6E-05			
	11	Gm08_44265646	C:T	0.20	0.7	4.8E-05	*Glyma08g44640* *Glyma08g44650*	AtVRN1 [4]	
	12	Gm08_44602360	G:A	0.21	0.8	5.6E-06			
	13	Gm08_44814503	C:T	0.23	0.7	5.2E-05	*Glyma08g45680*	FAR1 DNA-binding domain containing protein [5]	
	14	Gm09_4388948	T:C	0.19	0.9	5.4E-05			
	15	Gm09_6197309	G:A	0.18	0.8	1.9E-04			
	16	Gm09_6976053	A:G	0.13	1.0	2.1E-05	*Glyma09g07980*	AtELF8/AtVIP6 [6]	Ft 3-3
	17	Gm11_1395042	C:T	0.39	0.6	2.0E-04	*Glyma11g02230*	Nucleoporin-ralated	
	18	Gm11_17274491	T:C	0.12	1.2	2.1E-06	*Glyma11g20470*	Heparansulfate 6-o-sulfotransferase 3	
	19	Gm13_39699161	C:T	0.18	0.9	8.5E-05	*Glyma13g38975*	AtCLV3 [7]	
	20	Gm14_2918117	T:C	0.14	1.1	9.2E-05			
	21	Gm14_45475177	G:A	0.08	1.2	2.8E-05	*Glyma14g36221*	AtAGL16 [8]	Ft 3-1
	22	Gm18_48177289	C:A	0.09	1.4	1.3E-05			Fflr 10-2

	23	Gm18_60868392	T:C	0.19	1.0	1.4E-06			
	24	Gm19_40662679	T:G	0.34	-0.7	6.9E-05			Fflr 15-2, 16-4, 2-3, 4-3
	25	Gm20_276793	G:T	0.14	0.9	3.7E-05	*Glyma20g00440*	Jumonji domain containing protein [9]	
	26	Gm20_23754062	G:A	0.14	1.3	1.6E-06	*Glyma20g16880*	AtEMF2 [10]	FT 2-3
		Gm20_26991347	C:T	0.15	1.4	5.5E-08			
		Gm20_27852579	C:T	0.15	1.4	5.5E-08			
		Gm20_29208847	C:T	0.12	1.4	9.1E-08			
		Gm20_29983050	G:A	0.13	1.4	9.7E-08	*Glyma20g21082*	AtAGL6 [2]	
	27	Gm20_46730763	G:T	0.05	1.4	5.6E-05	*LOC100812369*	AP2/ERF and B3 domain-containing transcription factor [11]	
DTM	1	Gm11_10152581	T:C	0.08	-1.6	5.2E-06	*Glyma11g14150*	RNA-BINDING PROTEIN	
	2	Gm16_2412662	T:C	0.47	-0.9	2.5E-06	*Glyma16g02840*	AtLEW3 [12]	
	3	Gm16_2824073	T:G	0.11	-1.2	4.3E-06			
	4	Gm16_3006858	C:T	0.19	-1.2	3.8E-06	*Glyma16g03580*	Ring finger containing protein	
	5	Gm19_45000827	C:A	0.05	-2.3	7.6E-06	*Dt1*	AtTFL1 [13]	Pod mat 13-6
	6	Gm19_47909005	A:G	0.21	1.2	1.1E-05			
DFTM	1	Gm02_49988138	T:G	0.19	0.9	1.8E-05			
	2	Gm03_1597206	T:G	0.15	0.9	8.6E-05			
	3	Gm03_5316197	T:C	0.08	1.3	9.7E-05			
	4	Gm03_11220188	A:G	0.08	-1.2	1.5E-04			Pod mat 20-2
	5	Gm03_36330058	A:G	0.49	-0.9	7.3E-06			
	6	Gm04_35149844	C:T	0.25	-1.2	4.2E-07			Pod mat 18-3
	7	Gm06_10263943	C:A	0.06	-1.6	1.9E-05			
	8	Gm06_16167243	A:G	0.07	1.6	2.3E-05	*Glyma06g19820*	Betaine-aldehyde dehydrogenase	R3 2-1, R5 3-2, R7 2-1, Pod mat 25-2
	9	Gm08_1966701	G:T	0.18	-0.9	5.1E-05	*Glyma08g02881*	Unknown	

	10	Gm08_2636067	G:T	0.12	-1.0	8.3E-05			
	11	Gm09_6970117	T:G	0.14	-1.1	2.7E-05	*Glyma09g07980*	AtELF8/AtVIP6 [6]	Pod mat 16-3, 5-2
	12	Gm09_41155181	T:C	0.31	-0.8	3.5E-06			Pod mat 27-2
	13	Gm11_9332515	A:G	0.18	0.8	1.8E-05	*Glyma11g13090*	Serine-threonine protein kinase	Pod mat 17-2, 18-2
	14	Gm12_4363882	C:T	0.08	1.4	5.7E-06			
	15	Gm15_1609497	T:C	0.08	1.2	4.0E-05	*Glyma15g02390*	Mitochondrial processing peptidase	
	16	Gm16_2412662	T:C	0.47	-0.7	4.2E-05	*Glyma16g02840*	AtLEW3 [12]	
	17[f]	Gm16_2997247	C:T	0.11	-1.0	6.7E-06	*Glyma16g03570*	WRKY family transcription factor	
	18	Gm19_45255796	A:G	0.16	1.2	6.5E-05			R5 1-2, Pod mat 24-10, 24-4, 10-4, 13-6, 4-3
PH	1	Gm04_5175822	A:G	0.06	-11.5	1.0E-07			
	2	Gm06_14889674	C:T	0.19	-4.5	4.4E-05			
	3	Gm08_42349221	C:T	0.06	-7.2	1.3E-05			
	4	Gm09_9623583	T:G	0.21	3.6	8.8E-05			Plant height 18-3
	5	Gm09_40958920	C:T	0.08	-5.8	1.6E-04	*Glyma09g34601*	AtBRX [14]	
	6	Gm09_41147947	C:T	0.14	-5.1	1.3E-05			
	7	Gm10_7796028	A:C	0.09	6.6	7.2E-07	*Glyma10g08930*	GDSL-like Lipase	
	8	Gm11_6078943	C:A	0.06	-9.0	8.6E-06			Plant height 24-4
	9	Gm12_128224	T:C	0.12	-5.6	8.7E-05	*Glyma12g00320*	AtSAC1 [15]	
	10	Gm12_8911538	G:A	0.10	4.7	9.6E-05			
	11	Gm13_36042528	G:A	0.41	3.7	6.3E-06			Plant height 17-1
	12	Gm13_36823931	G:T	0.39	3.5	1.9E-05			Plant height 17-1
	13	Gm13_42933309	C:T	0.08	-6.9	8.2E-06			
	14	Gm14_48703687	G:T	0.28	3.7	1.4E-05			
	15	Gm15_3135042	G:A	0.06	-7.2	5.0E-06			

	16	Gm15_25719454	T:G	0.05	-7.6	1.6E-05			Plant height 26-10
	17	Gm16_5773005	G:A	0.07	-6.1	4.5E-05	*Glyma16g06333*	ASB1/TRP4/WEI7	
	18	Gm16_29597918	G:A	0.07	-10.8	1.0E-08	*Glyma16g25570*	AtHAM4 [16]	
	19	Gm17_40318849	A:G	0.38	-3.7	2.7E-05	*Glyma17g36350*	Unknown	
	20	Gm17_40538815	T:C	0.12	-6.2	5.8E-06			
	21	Gm18_58462762	A:C	0.22	-3.7	6.2E-05	*Glyma18g49080*	AtPIN1 [17]	Plant height 17-3, 25-5, 26-12, 33-2, 33-8, 34-2, 34-8
	22	Gm18_60516754	A:C	0.08	-7.2	4.0E-05			Plant height 26-12, 33-2, 34-2
	23	Gm19_8629858	G:A	0.05	-9.1	1.5E-06			
	24	Gm19_44389764	T:C	0.11	-10.9	3.7E-11	*Glyma19g37180*	Pectin lyase-like [18]	Plant height 1-1, 6-1 and 10-4
	25	Gm19_45000827	C:A	0.05	-16.0	3.0E-15	*Dt1*	AtTFL1 [13]	Plant height 4-2 and 13-8
	26	Gm20_376751	C:T	0.15	4.3	4.7E-05			
	27	Gm20_35951388	C:T	0.17	-4.4	7.5E-05			

[a] Start with the chromosome number and followed by physical position of the marker on that chromosome.
[b] MAF, minor allele frequency
[c] Respect to minor allele.
[d] Genes annotated in Glyma1.1, Glyma1.0, and NCBI RefSeq gene models in SoyBase (www.soybase.org) were used as the source of candidate genes.
[e] Based on the QTL list on SoyBase (www.soybase.org).
[f] The SNP was also detected significantly associated with DTM and was clumped in the locus DTM4.

References

1. Cao Y, Dai Y, Cui SJ, Ma LG: **Histone H2B Monoubiquitination in the Chromatin of FLOWERING LOCUS C Regulates Flowering Time in Arabidopsis**. *Plant Cell* 2008, **20**(10):2586-2602.
2. Yoo SK, Wu X, Lee JS, Ahn JH: **AGAMOUS-LIKE 6 is a floral promoter that negatively regulates the FLC/MAF clade genes and positively regulates FT in Arabidopsis**. *The Plant Journal* 2011, **65**(1):62-76.
3. Zhong XF, Dai X, Xv JH, Wu HY, Liu B, Li HY: **Cloning and expression analysis of GmGAL1, SOC1 homolog gene in soybean**. *Mol Biol Rep* 2012, **39**(6):6967-6974.
4. Levy YY, Mesnage S, Mylne JS, Gendall AR, Dean C: **Multiple roles of Arabidopsis VRN1 in vernalization and flowering time control**. *Science* 2002, **297**(5579):243-246.
5. Hudson M, Ringli C, Boylan MT, Quail PH: **The FAR1 locus encodes a novel nuclear protein specific to phytochrome A signaling**. *Gene Dev* 1999, **13**(15):2017-2027.
6. Oh S, Zhang H, Ludwig P, van Nocker S: **A mechanism related to the yeast transcriptional regulator Paf1c is required for expression of the Arabidopsis FLC/MAF MADS box gene family**. *Plant Cell* 2004, **16**(11):2940-2953.
7. Clark SE, Running MP, Meyerowitz EM: **Clavata3 Is a Specific Regulator of Shoot and Floral Meristem Development Affecting the Same Processes as Clavata1**. *Development* 1995, **121**(7):2057-2067.

8. Alvarez-Buylla ER, Liljegren SJ, Pelaz S, Gold SE, Burgeff C, Ditta GS, Vergara-Silva F, Yanofsky MF: **MADS-box gene evolution beyond flowers: expression in pollen, endosperm, guard cells, roots and trichomes**. *Plant J* 2000, **24**(4):457-466.

9. Noh B, Lee SH, Kim HJ, Yi G, Shin EA, Lee M, Jung KJ, Doyle MR, Amasino RM, Noh YS: **Divergent roles of a pair of homologous jumonji/zinc-finger-class transcription factor proteins in the regulation of Arabidopsis flowering time**. *Plant Cell* 2004, **16**(10):2601-2613.

10. Yoshida N, Yanai Y, Chen LJ, Kato Y, Hiratsuka J, Miwa T, Sung ZR, Takahashi S: **EMBRYONIC FLOWER2, a novel polycomb group protein homolog, mediates shoot development and flowering in Arabidopsis**. *Plant Cell* 2001, **13**(11):2471-2481.

11. Jofuku KD, Denboer BGW, Vanmontagu M, Okamuro JK: **Control of Arabidopsis Flower and Seed Development by the Homeotic Gene Apetala2**. *Plant Cell* 1994, **6**(9):1211-1225.

12. Zhang M, Henquet M, Chen ZZ, Zhang HR, Zhang Y, Ren XZ, van der Krol S, Gonneau M, Bosch D, Gong ZZ: **LEW3, encoding a putative alpha-1,2-mannosyltransferase (ALG11) in N-linked glycoprotein, plays vital roles in cell-wall biosynthesis and the abiotic stress response in Arabidopsis thaliana**. *Plant J* 2009, **60**(6):983-999.

13. Liu B, Watanabe S, Uchiyama T, Kong F, Kanazawa A, Xia Z, Nagamatsu A, Arai M, Yamada T, Kitamura K *et al*: **The soybean stem growth habit gene Dt1**

is an ortholog of Arabidopsis TERMINAL FLOWER1. *Plant Physiol* 2010, **153**(1):198-210.

14. Beuchat J, Li S, Ragni L, Shindo C, Kohn MH, Hardtke CS: **A hyperactive quantitative trait locus allele of Arabidopsis BRX contributes to natural variation in root growth vigor**. *Proc Natl Acad Sci U S A* 2010, **107**(18):8475-8480.

15. Zhong R, Burk DH, Nairn CJ, Wood-Jones A, Morrison WH, 3rd, Ye ZH: **Mutation of SAC1, an Arabidopsis SAC domain phosphoinositide phosphatase, causes alterations in cell morphogenesis, cell wall synthesis, and actin organization**. *Plant Cell* 2005, **17**(5):1449-1466.

16. Engstrom EM, Andersen CM, Gumulak-Smith J, Hu J, Orlova E, Sozzani R, Bowman JL: **Arabidopsis homologs of the petunia hairy meristem gene are required for maintenance of shoot and root indeterminacy**. *Plant Physiol* 2011, **155**(2):735-750.

17. Sassi M, Lu Y, Zhang Y, Wang J, Dhonukshe P, Blilou I, Dai M, Li J, Gong X, Jaillais Y: **COP1 mediates the coordination of root and shoot growth by light through modulation of PIN1-and PIN2-dependent auxin transport in Arabidopsis**. *Development* 2012, **139**(18):3402-3412.

18. Derbyshire P, McCann MC, Roberts K: **Restricted cell elongation in Arabidopsis hypocotyls is associated with a reduced average pectin esterification level**. *BMC plant biology* 2007, **7**(1):31.
